# Palynological data of cores MSM5/5–712–2 and PS2863/1–2 from northeastern Fram Strait spanning the last glacial maximum to present

**DOI:** 10.1016/j.dib.2019.103899

**Published:** 2019-04-05

**Authors:** Jade Falardeau, Anne de Vernal, Robert F. Spielhagen

**Affiliations:** a GEOTOP-UQAM, CP 8888, Montréal, H3C 3P8, Canada; b GEOMAR Helmholtz Centre for Ocean Research, 24148, Kiel, Germany; c Academy of Sciences, Humanities and Literature, 55131, Mainz, Germany

**Keywords:** Fram strait, Last glacial maximum, Late and postglacial, Holocene, Palynology, Temperature, Salinity, Sea-ice, Dinocysts

## Abstract

The palynogical data of two sites from northeastern Fram Strait (MSM5/5–712 and PS2863) encompassing the last 23,000 years are presented here. The data set first includes the palynomorph concentrations: dinocysts (cysts/g) and their fluxes (cysts/cm^2^/yr) as well as pollen grains, spores, organic linings, *Halodinium*, reworked palynomorphs and *Pediastrum* represented in #/g. It also includes the relative abundance (%) of dinocyst taxa at sites MSM5/5–712 and PS2863. Finally, this Data in Brief comprises reconstructions of sea-surface conditions at the two sites, which include sea-surface temperature (°C) in summer and winter, sea-surface salinity (psu) in summer and winter, sea-ice cover (month/yr) and productivity (gC/m^2^yr). The most probable values in addition to minimum and maximum possible are reported. The data is presented in function of the cores depth and age. For more details on this data and the chronology of the cores, see [1].

**Table:** Specifications table

Subject area	*Earth Sciences*
More specific subject area	*Paleoceanography*
Type of data	*Tables*
How data was acquired	*Microscope Leitz Aristoplan at 400X magnification* *R version 3.4.3 (to run the modern analog technique)*
Data format	*Analyzed (the data presented results from concentration and flux calculations (see Experimental Design, Materials and Methods section below) and the modern analog technique*[2]*based on the raw counts available in**Ref. [3]**.)*
Experimental factors	*The MSM5/5-712-2 core was retrieved on the MSM05/5b expedition of the RV Maria S. Merian**[4]**and subsampled at 4 cm intervals from 0 to 283 cm and at 8 cm interval down to 777 cm.* *The PS2863/1–2 cores were retrieved during the ARK-XIII/2 expedition of the RV Polarstern**[5]**and were combined to form a composite core (PS2863). The first 41 cm were subsampled over slices of 1 cm thick at 1 cm interval (represented by the box core PS2863-2) and at 4 cm interval from 41 to 184 cm (represented by the gravity core PS2863-1).*
Experimental features	*For microscope observations and counting of palynomorphs, the sediment samples were sieved and treated with hydrofluoric and hydrochloric acids in order to concentrate the refractory organic matter in the 10–106 μm size fraction. The raw counts were then subjected to basic calculations to obtain the palynomorph's concentration and flux. The sea-surface conditions were obtained based on the modern analog technique*[2].
Data source location	*MSM5/5-712-2: 78°54.94′N, 6°46.04′E* *PS2863/1–2: 80°33.46′N, 10°17.96′E*
Data accessibility	*The data is available within this article.*
Related research article	*Falardeau, J., de Vernal, A., Spielhagen, R.F. Paleoceanography of northeastern Fram Strait since the last glacial maximum: Palynological evidence of large amplitude changes. Quaternary Science Reviews. 2018,195:133-152*[1].

**Table:** 

**Value of the Data** • The data available from this document include 2 continuous records of estimated sea-surface parameters spanning the last 23,000 years. These data can be used for model-data comparison of past hydrographic conditions in western and northwestern Svalbard. • The palynological data from core MSM5/5–712-2 can be used for regional proxy comparisons with other records of past hydrographic conditions, including biomarkers, planktic and benthic foraminifer assemblages, stable isotope data or sedimentological properties, for example. • This data can serve for palynologists to compare dinocyst assemblages in different settings (time and space).

## 1 Data

Three different kinds of data derived from the raw counts of palynomorphs at sites MSM5/5–712 and PS2868 located in northeastern Fram Strait [3], northwest of Svalbard, Norway, are shown in this Data in Brief article in the form of 6 excel sheets. The calculated concentrations (#/g) of dinocysts and their fluxes (#/g/cm^2^) and concentrations of other palynomorphs: spores, pollen grains, organic linings of benthic foraminifers, *Halodinium*, reworked palynomorphs and *Pediastrum* in the sediments of sites MSM5/5–712 and PS2863, respectively, are presented in the first two tables. Then, the dinocyst assemblages (relative abundances of taxa in %) in the samples from each site are shown in the following two tables. Finally, quantitative reconstructions of sea-surface conditions (sea-surface temperature (°C) in summer and winter, sea-surface salinity (psu) in summer and winter, sea-ice cover (month/yr) and productivity (gC/m^2^yr)) at sites MSM5/5–712 and PS2863 can be found in the last two tables. The original maximum, minimum and most probable values derived from the reconstructions are reported as well (no additional corrections were applied). All data are presented against age and depth in the cores (according to the age models described in Ref. [1]). Details on all the calculations can be found in the next section.

### 1.1 Experimental design, materials and methods

For each sample, about 5 cm^3^ of sediment were wet sieved and one capsule of the marker grain *Lycopodium clavatum* (the number of spores in one capsule is known) was added for the palynomorph concentration calculations [6]. In order to preserve the organic matter only, the 10–106 μm fraction was subjected to three hydrochloric acid treatments to dissolve carbonate particles interspersed by hydrofluoric acid treatments to dissolve the silica particles. The residues were mounted on slides in glycerin jelly and observed under microscope at 400X optical magnification for palynological analysis. Minimum counts of 300 dinocysts per sample were targeted.

Once the counting completed, the palynomorph concentrations in #/g were calculated as follows:

(1) Np = (Ne × np)/ne

Np: Total number of the palynomorphs

Ne: Known number of *L. clavatum* spores

np: Number of palynomorphs counted

ne: Number of *L. clavatum* spores counted

(2) Palynomorph concentrations (#/g) = Np/dry sediment weight (g)

The fluxes were calculated as follows:

(1) Np/volume (cm^3^) = Dinocyst concentrations (#/cm^3^)
(2) Flux (#/cm^2^/yr) = Sedimentation rate (cm/yr) × Concentration (#/cm^3^)

Reconstructions of sea-surface conditions were obtained based on the modern analog technique (MAT; [2]) applied to the dinocyst relative abundances according to Ref. [7]. The MAT approach consists of identifying the 5 best analogs from the dinocyst spectra (relative abundance of each taxa) of the surface sediment data set (modern assemblages) that includes 1776 sites [7], [8]. The sea-surface condition values represent the average of the 5 selected analogs weighted inversely to the distance. The maximum and minimum are determined based on the minimum and maximum values from the set of analogs.
